# Emergency Physician Performed Ultrasound for DVT Evaluation

**DOI:** 10.1155/2011/938709

**Published:** 2011-03-06

**Authors:** John Christian Fox, Kiah Christine Bertoglio

**Affiliations:** Department of Emergency Medicine, Irvine Medical Center, University of California, 101 The City Drive South, Route 128-01, Orange, CA 92868, USA

## Abstract

Deep vein thrombosis is a common condition that is often difficult to diagnose and may be lethal when allowed to progress. However, early implementation of treatment substantially improves the disease prognosis. Therefore, care must be taken to both acquire an accurate differential diagnosis for patients with symptoms as well as to screen at-risk asymptomatic individuals. Many diagnostic tools exist to evaluate deep vein thrombosis. Compression ultrasonography is currently the most effective diagnostic tool in the emergency department, shown to be highly accurate at minimal expense. However, limited availability of ultrasound technicians may result in delayed imaging or in a decision not to image low-risk cases. Many studies support emergency physiciansas capable of accurately diagnosing deep vein thrombosis using bedside ultrasound. Further integration of ultrasound into the training of emergency physicians for use in evaluating deep vein thrombosis will improve patient care and cost-effective treatment.

## 1. Introduction

Deep vein thrombosis (DVT) is a life threatening condition affecting approximately 1%-2% of Americans, from which development of pulmonary embolism (PE) is the primary concern. The condition exists as a spectrum from DVT to PE and may be referred to as venous thromboembolism (VTE). Each year in the United States, approximately 2 million people develop DVT, and 300,000 people die from PE [1, 2]. Once identified, many treatment strategies are available that substantially improve the disease prognosis [3]. However, it is estimated that only one-third of VTE cases are detected [4]. Therefore, focus on early identification is critical for patient outcome. Although many diagnostic tools are available to evaluate the presence of DVT, the use of ultrasonography (US) for routine DVT evaluation is superior in accuracy, cost, and feasibility.

A large number of VTE diagnoses are made in the emergency department (ED). A recent review of data from the National Ambulatory Medical Care Survey (NAMC) and National Hospital Ambulatory Medical Care Survey (NHAMCS) from 1997 to 2006 found an average of 236,000 ED cases of DVT annually and a trend towards an increasing prevalence of venous disease [5]. However, the use of ultrasound to diagnose DVT in the ED has not exceeded 30% since 1999. Furthermore, there was no significant change in use of US for DVT diagnoses over the decade analyzed [5]. Despite the wide acceptance in the recent literature of US for determining DVT diagnosis in the ED [6], controversy still seems to exist on its use by emergency physicians as standard of care, US training remains variable between emergency physicians, and reported data indicates that it is utilized in less than one-third of diagnoses [5]. 

## 2. Patient Presentation

DVT may be difficult to diagnose as symptoms of the condition also occur in a variety of other disorders, and approximately half of patients with DVT are asymptomatic [7]. Symptoms of DVT include swelling, pain, tenderness, warmth, and prominent superficial veins on the affected limb; symptoms of PE include chest pain, dyspnea, cough, and syncope, which may be accompanied by tachypnea, crepitation, tachycardia, and hypotension. Therefore, it is important to consider risk factors for DVT such as age, previous DVT, extended bed rest, pregnancy, use of oral contraceptive, hormone replacement therapy, recent surgery, active malignant neoplasm with or without concurrent chemotherapy, neurological disease with paresis extremity, and trauma when determining differential diagnoses. An individual's risk of DVT may be estimated by the Wells Score which provides means to calculate a weighted composite of the numerous risk factors into a pretest probability of low, moderate, and high risk of DVT, indicating a 3%, 17%, and 75% probability of identifying DVT, respectively [8].

Pulmonary embolism is found in approximately half of patients with documented DVT [1], from which at least 90% originate from DVT in the lower extremities [6] and approximately 8% originate from DVT in the upper extremities [7]. Thrombi found in the common femoral, superficial femoral, and popliteal veins pose the greatest threat to causing a PE. Veins distal to the popliteal vein often independently dissolve and are rarely an origin of a PE [9]. The remaining thrombi causing PE originate in other veins such as deep pelvic, renal, and inferior vena cava. 

## 3. Emergency Physician-Performed US

Due to the high incidence of trauma and critical conditions (both of which are risk factors for DVT) in ED patients, VTE must at least be considered as a possible primary or concomitant pathology in a majority of admittances. An estimated two-third of individuals with VTE are unidentified [4]. Furthermore, DVT is only found in approximately 25% of patients with suggestive symptoms as many other disorders present with similar manifestations [10]. The requirement of imaging to eliminate the possibility of DVT and the large number of undiagnosed DVT cases provides substantial support for extensive patient screening for DVT. With a sensitivity and specificity >95% for both upper extremity [11] and proximal lower extremity thrombi [12], bedside US is the least invasive, most accurate, and fastest method of evaluating DVT at the lowest cost.

Ultrasound technicians have limited availability in many hospitals, especially during the night, which frequently causes delays in imaging and may even result in the decision not to US make symptomatic patients who are not high risk for VTE. US trained emergency physicians are able to quickly scan and interpret images during their initial evaluation without the additional expense and time US technicians require. It also allows instantaneous discovery of other potential pathologies including cellulitis, abscess formation, Baker's cyst, hematoma, and edema [7]. Therefore, emergency physician bedside scanning provides the most cost-efficient method for delineating diagnosis of suspected DVT. Many studies have shown that emergency physicians are capable and effective in performing US scanning of DVT [12]. However, emergency physician training on US remains variable across the country, and the use of US to identify DVT is reported in less than one-third of the cases [5]. More uniform training of emergency physicians in US technique and standardized use of US to screen for DVT in the ED is likely to lead to increased early identification of DVT, lower costs, and overall improved patient care. 

## 4. Other Diagnostic Tools

In addition to US, the presence of DVT may be evaluated by a variety of other diagnostic tools including D-dimer test, contrast venography, impedance plethysmography, computed tomography (CT), and magnetic resonance imaging (MRI). The D-dimer test, a measurement of fibrin degradation product in the blood, is generally accepted to be reliable in ruling out DVT but is not able to confirm the presence of a thrombosis. Therefore, it is typically used in conjunction with US as a screening test to determine if imaging is required. It is important to note that patients with a negative CUS and a positive D-dimer test should have a repeat CUS within the following week if they are at high risk for DVT. However, the value of the D-dimer test may decline after the first week of thrombolytic onset, warranting US imaging in patients even with a negative D-dimer test if symptoms have persisted longer than a week or for high-risk patients [13].

Contrast venography was previously the gold standard for DVT evaluation and may still be utilized if other techniques are inconclusive. Although effective, the technique is invasive, expensive, time consuming, requires specialized personnel, and may introduce a variety of complications including iatrogenic DVT [7]. Although impedance plethysmography is noninvasive, its sensitivity may be as low as 20% and therefore is not frequently used for diagnosing proximal DVT [14]. Magnetic resonance imaging (MRI) is an accurate and harmless alternative to US, but it remains costly with low availability and has a variety of exclusions including patients with pacemakers [7]. In addition to their expense and limited availability, CT scans impose extensive radiation on the patient and should be reserved for circumstances that necessitate their use. 

## 5. Ultrasound Technique

Limited compression ultrasound (CUS) and duplex ultrasonography are both US methods available to evaluate DVT. CUS is the primary US technique used to identify DVT and is accomplished by imaging with and without compression, a process that assesses the collapsibility of veins and may be rapidly performed. Lack of compressibility is the main determinant of a DVT. Duplex ultrasonography is a more comprehensive examination involving the use of color flow and Doppler techniques, requiring about 45 minutes to complete. Duplex ultrasonography is not typically used for DVT evaluation as CUS is equally accurate and may be completed within minutes. However, the use of color and spectral Doppler can assist in the CUS examination when the image is difficult to obtain in instances such as morbidly obese patients or in those with unusual anatomy. Doppler is useful in differentiating venous from arterial flow. Arteries are identified by observing pulsatile flow with color Doppler and the presence of an arterial waveform with spectral Doppler, as opposed to continuous flow and a venous waveform seen in venous structures. An augmentation test may be used, in which squeezing a distal portion of a vein should cause an increased blood flow in its proximal portion. Lack of augmentation suggests that a clot exists somewhere between the point of compression and the ultrasound probe. However, recent evidence suggests that only in rare cases does augmentation add pertinent information that is not obtained from gray-scale imaging [15]. Therefore, in routine imaging, emergency physician-performed CUS should only add minutes to their examination. 

**Figure 1:**
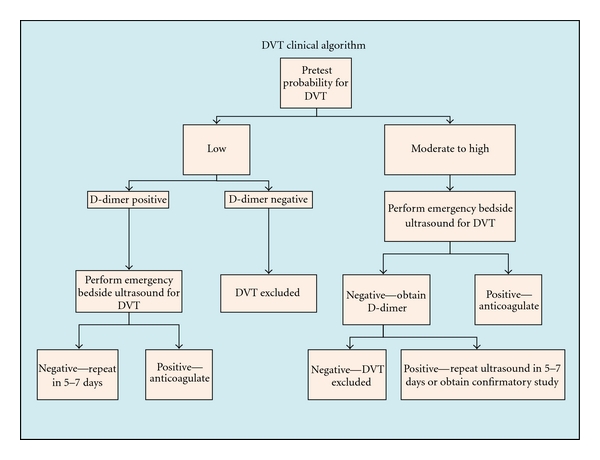
DVT clinical algorithm suggested by the American College of Emergency Physicians (ACEP) [17].

CUS may be performed utilizing a whole-leg or 2-point strategy. In whole-leg CUS, both proximal and distal veins are evaluated. Two-point CUS only involves compression of the common femoral vein (CFV) and popliteal vein (PV) to their branching points. Studies have indicated that the whole-leg and 2-point strategies are equivalently effective in identifying DVT, and thrombi with potential to form PE are found in the CFV and/or PV. Therefore, emergency physicians should perform 2-point CUS and instruct patients with thrombi found distal to the popliteal vein, high pretest patients, and patients with positive D-dimer and negative CUS to consult their primary care physician and schedule a follow-up ultrasound within the week. In the event that a patient is unable to consult a physician in the necessary time frame, they should return to the ED within the week (Figure 1) [16]. 

## 6. Image Acquisition with 2-Point CUS

Capturing a quality image is assisted by positioning the patient so that the veins of the leg are distended, which may be accomplished by the reverse Trendelenburg position in supine patients. Imaging is focused on the CFV and PV to their bifurcations. Using a high-frequency linear transducer, imaging should begin in a transverse orientation at the confluence of the CFV with the greater saphenous vein in the inguinal region. The CFV is identified medial to the common femoral artery (CFA). Pressure applied with the probe should cause collapse of the veins. Even though the greater saphenous vein is a superficial vessel, thrombus at the saphenofemoral junction has a high risk of embolizing, and patients with such conditions should be treated with anticoagulation. Moving the probe distally, both the CFA and the CFV will bifurcate into deep and superficial branches. The deep femoral vein runs deep in the proximal thigh musculature and is not routinely imaged as it does not impose a high risk for PE. As scanning continues down the thigh, the femoral vein will transition from its position medial to the artery to posterior to the artery, appearing below the artery on the ultrasound image. Venous structures may be more easily compressed by reverse compression within Hunter's canal. Rather than compressing the probe into the leg, the probe is held steady, and the nonscanning hand is used to compress the leg into the probe. The probe should then be placed posteriorly in the popliteal fossa to follow the popliteal vein (PV) with compressions every 1-2 cm until its trifurcation distally. This probe orientation places the vein closer than the artery to the probe, creating the appearance of the vein being “on top” of the artery. Examination of the PV is assisted when the patient's knee is slightly flexed and with external rotation of the hip. 

## 7. Limitations

Ultrasonography is limited in efficacy of detecting calf, pelvic, and abdominal thrombi in addition to circumstances that compromise the ability to obtain a high-quality image. The sensitivity of US declines for calf thrombi to as low as 73% in symptomatic patients and 50% in asymptomatic patients [8]. Thrombi found distal to the popliteal vein should be reimaged in 3–5 days to ensure that it has not embolized to more proximal veins. 

Although it is less common for PE to originate from pelvic or abdominal thrombi, other diagnostic techniques must be used if there is concern for possible clots in these areas as they cannot be reliably identified by US. Other diagnostic tools may also be needed in cases in which an adequate image is not able to be obtained, such as in obese patients or in patients who are unable to sustain compression of the probe. Although the sensitivity and specificity of US is >95%, patients at very high risk for VTE should have additional imaging tests even with a normal US in order to confirm that they are negative for DVT. 

## 8. Conclusions

There are many benefits to utilizing CUS for evaluating potential cases of DVT including high accuracy with minimal time and expense. The CUS technique provides means for the emergency physician to rapidly screen patients at bedside for DVT, through which process other pathologies may be identified. Despite these advantages, statistics indicate that US is not being utilized in the ED in more than one-third of DVT diagnoses. Emergency physicians are capable of accurately diagnosing DVT using bedside US. Further integration of US into the training of emergency physicians and routine treatment of DVT will improve patient care as well as time and cost of treatment. Other diagnostic tools should be used in circumstances of suspected calf, pelvic, and abdomen clots or in situations where obtaining an image through US is compromised.
